# Role of procalcitonin as a predictor in difficult laparoscopic cholecystectomy for acute cholecystitis case: A retrospective study based on the TG18 criteria

**DOI:** 10.1038/s41598-019-47501-0

**Published:** 2019-07-29

**Authors:** Tianchong Wu, Minjun Luo, Yuehua Guo, Jiangang Bi, Yusheng Guo, Shiyun Bao

**Affiliations:** 1 Department of Hepatobiliary and Pancreatic Surgery, The Second Medical College, Shenzhen People’s Hospital, Jinan University, Shenzhen, 518020 Guangdong Province China; 2 Department of Operation room, The Second Medical College, Shenzhen People’s Hospital, Jinan University, Shenzhen, 518020 Guangdong Province China

**Keywords:** Experimental models of disease, Cholelithiasis, Cholecystitis

## Abstract

Difficult laparoscopic cholecystectomy (DLC) is difficult to precisely predict before operation. This observational cohort study aimed to evaluate the predictive value of procalcitonin (PCT) for DLC in patients with acute cholecystitis (AC). A total of 115 patients were included in the study from January 2017 to April 2018. Multiple logistic regression and receiver-operating characteristic (ROC) were performed to evaluate the predictive value of PCT levels in DLC. Patients with DLC had significantly higher Tokyo Guidelines 2018 (TG18) grade (*P* = 0.002) and levels of C-reactive protein (CRP) (*P* = 0.007) and PCT (*P* < 0.001). The cut-off value of PCT for predicting DLC was 1.50 ng/ml. The sensitivity and specificity were 91.3% (95% CI 78.3–97.1) and 76.8% (95% CI 64.8–85.8), respectively. The area under ROC curve was 92.7% (95% CI 88.2–97.3, *P* < 0.001). Our results suggested that PCT was a good predictor for DLC in the AC patients, but further research is necessary. Monitoring of PCT trends in AC patients may be useful for preoperative risk assessment.

## Introduction

According to the Tokyo Guidelines 2018 (TG18), laparoscopic cholecystectomy (LC) is a top-priority treatment for acute cholecystitis (AC)^1^. Difficult laparoscopic cholecystectomy (DLC) is a primal problem which surgeons may encounter when treating AC. DLC has different definitions, but the conversion rate of laparotomy and iatrogenic injury rate are often regarded as important indicators for defining DLC^2^. Accurate prediction of DLC can help surgeons to prepare for perioperative challenges, optimize surgical procedures and reduce the postoperative complications. However, there are just a few scoring systems to assess the risk of LC to convert to open cholecystectomy for AC, but they offer no effective prediction of DLC^3–5^. In addition, some studies included cases with acute/chronic cholecystitis, pancreatitis, cholangitis, or choledocholithiasis^2,6,7^.

In the preoperative planning of AC, the severity of AC and the prediction of DLC should be considered^2^. Previously, the severity of AC was predicted by assessing leukocytosis and C-reactive protein (CRP) levels according to the Tokyo Guidelines, but neither of them could be utilized to predict the DLC^8^. Procalcitonin (PCT) has been used as an important indicator for early diagnosis of AC in some retrospective studies, although the diagnostic value of PCT for AC is still needed to be further validated in the TG18^8,9^. In addition, the levels of CRP in the initial 6~12 h of AC is usually within the normal range. On the contrary, plasma levels of PCT increased significantly in the first hour of AC-induced systemic inflammatory response, and peaked significantly earlier than that of CRP^9,10^.

Therefore, we hypothesized that preoperative PCT levels may have the predictive value for DLC in AC patients. The application of PCT in the preoperative assessment of AC patients may be valuable in improving the accuracy of AC diagnosis and predicting DLC. The purpose of this study is to evaluate the predictive of PCT for DLC in AC patients.

## Methods and Patients

This was a retrospective cohort study involving all consecutive AC patients receiving LC surgery in The Second Medical College of Jinan University, Shenzhen People’s Hospital from January 2017 to April 2018. Patients aged older than 18 years, diagnosed with AC based on TG18 and undergoing LC (from onset to surgery less than 96 hours) were included in the current study. Patients with any immunosuppressive state, uncontrolled amalgamation (diabetes, autoimmune disease, and liver failure), pregnancy, previous antibiotics, anticoagulants or anti-inflammatory drugs were excluded. Patients suffering from other acute biliary and pancreatic diseases, such as cholangitis, pancreatitis, choledocholithiasis and Mirizzi syndrome, were also excluded. LC was the primary treatment option for all patients in this study. All of the surgical operations were completed by the same surgical team, which has more than 20 years of clinical experience in hepatobiliary and pancreatic surgery and more than 1000 cases of LC. This study was approved by the ethics committee of the Shenzhen People’s Hospital and was conducted in accordance with the Declaration of Helsinki. Written informed consent was obtained from all patients.

Baseline variables included age, gender, body mass index (BMI) kg/m^2^, and previous abdominal operations, American Society of Anesthesiologists (ASA) score^11^, comorbidities. Clinical data included body temperature (°C), right upper quadrant (RUQ) rebound tenderness, Murphy’s sign, and duration of symptoms. Pre-operational laboratory examination included total leukocyte count (10^9/L), liver function test, CRP (mg/dl) and PCT (ng/ml). The gallbladder (GB) dimensions, GB thickness, cholecystolithiasis incarceration, and pericholecystic fluid collection were measured by ultrasonography. The severity of AC in all patients was graded according to the TG18 diagnostic criteria^8^. Operative time, intraperitoneal adhesion, intraoperative bleeding, the type of cholecystectomy, Clavien-Dindo classification of surgical complications^12^, timing and mode of cholecystectomy and final diagnosis were reported as operative findings and postoperative outcomes. The data was reported according to the STROCSS guidelines for cohort study^13^.

DLC was defined as a total operation time of more than 120 minutes, more than 40 minutes to obtain a critical view of safety (CVS) or be converted to open surgery because of the difficulties of laparoscopic surgery or complications (bleeding). On the contrary, LC without these features was defined as nondifficult LC (NDLC). According to the above definition, patients were divided into two groups: DLC and NDLC. Postoperative complications were scored according to Clavien-Dindo classification^12^.

### Statistical analysis

Continuous data was expressed as the mean ± standard ($\bar{\chi }$ ± S) and examined by *t* test or the Mann–Whitney *U* test based on distribution, and the categorical data was presented as number (%) and compared by *Chi-square* test or Fisher exact test (if expected value < 5 was observed). Predictive factors for DLC were identified by univariate and multivariate logistic regression analyses. The significant independent variable in univariate results would be included in the multivariate model. The variables significant in both univariate and multivariate results would be recognized as predictive factors for DLC. Receiver-operating characteristic (ROC) curve analysis was performed, and areas under the curve (AUC), sensitivity, specificity and their 95% confidence interval (CI) were reported. The cut-off of continuous variable (except for BMI) for the dichotomous group in the logistic regression analyses was suggested by comparatively maximum Youden’s index. BMI was grouped by Chinese BMI standard^14^. Except power analysis, all analyses were performed using IBM SPSS Version 22 (SPSS Statistics V22, IBM Corporation, Somers, New York). The statistical significance level for all the tests was set at a *P* < 0.05, two-tailed. The statistical power analysis of the 2 × 2 contingency table yielded by cut-off was analyzed using software G*Power version 3.1 (Heinrich-Heine-Universität Düsseldorf, Düsseldorf, German) to examine the post-hoc power of diagnostic results. In power analysis, the proportion p1 and p2 were corresponding to the ratios of patients who meet the cut-off in DLC and NDLC groups, i.e., sensitivity and 1-specificity.

## Results

**Figure 1:**
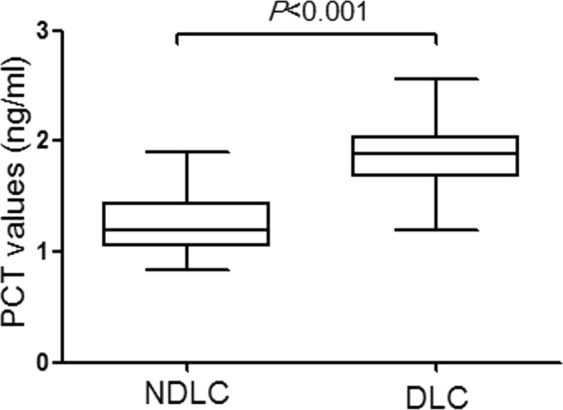
Box & Whisker Plot for PCT values in the DLC group and NDLC group showed that PCT values were significantly increased in the DLC group (*P* < 0.001). The transverse lines in the boxes represent the median values; the upper and lower boundaries of the boxes represent the 25th and 75th percentiles; the upper and lower poles outside the boxes represent the 90th and 10th percentiles.

From January 2017 to April 2018, 115 patients with AC were retrospectively included. Of them, 40% cases (n = 46) and 60% cases (n = 69) were grouped into the DLC and NDLC group, respectively. The preoperative data (demographic, clinical, laboratory, ultrasound and TG18 severity grading) were compared between the two groups (Table 1). The classification of DLC group had significantly higher TG18 grade (Grade ≥ II, DLC vs. NDLC; 87.0% vs. 59.4%, *P* = 0.002), levels of CRP (DLC vs. NDLC; 117.7 ± 13.0 mg/L vs. 100.4 ± 49.1 mg/L, *P* = 0.007) and PCT (DLC vs. NDLC; 1.9 ± 0.3 ng/ml vs. 1.3 ± 0.3 ng/ml *P* < 0.001) (Fig. 1). The incidence of cholecystolithiasis incarceration (73.9% vs. 50.7%, *P* = 0.013) and peri gallbladder effusion (45.7% vs. 18.8%, *P* = 0.004) was significantly higher in the DLC group than in the NDLC group.

**Table 1:** Clinical characteristics of preoperative.

	DLC (n = 46)	NDLC (n = 69)	*P*-value
Gender(female/male)	18:28	29:40	0.757
Age (y), mean (SD)	56.3 ± 15.3	51.7 ± 13.4	0.092
BMI (kg/m^2^), mean (SD)	22.3 ± 3.1	22.5 ± 2.9	0.713
Previous laparotomy, n (%)	8 (17.4)	10 (14.5)	0.675
ASA, n (%)			
I	36 (78.3)	48 (69.6)	0.668
II	7 (15.2)	14 (20.3)	
III	2 (4.3)	6 (8.7)	
IV.V	1 (2.2)	1 (1.4)	
Comorbidity, n (%)			
Hypertension	12 (26.1)	14 (20.3)	0.098
Diabetes mellitus	13 (28.3)	12 (17.4)	
Coronary artery disease	2 (4.3)	2 (2.9)	
Liver cirrhosis	7 (15.2)	7 (10.1)	
Chronic obstructive pulmonary disease	1 (2.2)	0 (0.0)	
Clinical data			
Body temperature (°C), mean (SD)	37.9 ± 0.7	37.8 ± 0.8	0.302
RUQ rebound tenderness, n (%)	44 (95.7)	60 (87.0)	0.195
Murphy’s sign, n (%)	43 (93.5)	58 (84.1)	0.130
Duration of symptoms (day), mean (SD)	2.9 ± 0.9	2.8 ± 1.0	0.719
Laboratory data, mean (SD)			
WBC (10^9/L)	12.6 ± 2.6	12.5 ± 2.5	0.847
TB (μmol/L)	30.3 ± 8.9	29.1 ± 9.7	0.506
DB (μmol/L)	14.5 ± 4.3	13.3 ± 4.4	0.135
ALT (U/L))	52.4 ± 23.5	46.7 ± 27.9	0.259
AST (U/L)	48.1 ± 22.3	47.2 ± 25.6	0.843
CPR (mg/L)	117.7 ± 13.0	100.4 ± 49.1	0.007
PCT (ng/ml)	1.9 ± 0.3	1.3 ± 0.3	<0.001
Ultrasonic diagnosis			
GB long axis (cm), mean (SD)	9.5 ± 1.4	9.2 ± 1.3	0.703
GB transverse (cm), mean (SD)	4.4 ± 0.8	4.1 ± 0.7	0.909
GB thickness (mm), mean (SD)	4.8 ± 0.8	4.4 ± 0.7	0.089
Cholecystolithiasis incarceration, n (%)	34 (73.9)	35 (50.7)	0.013
Peri gallbladder effusion, n (%)	21 (45.7)	13 (18.8)	0.004
TG18 severity grading, n (%)			
Grade I	6 (13.0)	28 (40.6)	0.002
Grade II	36 (78.3)	38 (55.1)	
Grade III	4 (8.7)	3 (4.3)	

Data are indicated n (%) or $\bar{\chi }$ ± S.

Compared with the NDLC group, the DLC group had more blood loss during operation (70.9 ± 33.9 ml vs. 15.9 ± 9.9 ml, *P* < 0.001) and longer operation time (DLC vs. NDLC; 147.4 ± 17.9 min vs. 66.8 ± 13.4 min, *P* < 0.001). The incidence of gangrenous cholecystitis in DLC group was higher than in NDLC group (34.8% vs. 1.4%, *P* < 0.001), while, simple cholecystitis was more common in NDLC group than in DLC group (2.2% vs. 29%, *P* < 0.001). Only one patient in the DLC group was switched to open surgery due to severe abdominal adhesion and intraoperative bleeding. No bile duct injury occurred in all patients. Based on the Clavien-Dindo classification, DLC group had a higher incidence of postoperative complications (DLC vs. NDLC: 17.4% vs. 2.9%, *P* = 0.024). Four patients in the DLC group needed subtotal cholecystectomy, but none in the NDLC group (*P* = 0.024). The comparison of intraoperative variables between the two groups was shown in Table 2.

**Table 2:** Operative findings and postoperative outcomes.

	DLC (n = 46)	NDLC (n = 69)	*P*-value
Operative time(min), mean (SD)	147.4 ± 17.9	66.8 ± 13.4	<0.001
Dense adhesions, n (%)	20 (43.5)	15 (21.7)	0.013
Blood loss (ml)	70.9 ± 33.9	15.9 ± 9.9	<0.001
Operation opportunity, n (%)			
Emergency operation	40 (87.0)	41 (59.4)	0.002
Selective operation	6 (13.0)	28 (40.6)	
Conversion, n (%)	1 (2.2)	0 (0.0)	0.400
Subtotal cholecystectomy, n (%)	4 (8.7)	0 (0.0)	0.024
Clavien–Dindo classification, n (%)			
Mild–moderate (grade I–II)	7 (15.2)	2 (2.9)	0.024
Severe (grade III–V)	1 (2.2)	0 (0.0)	
Final diagnosis, n (%)			
Acute simple cholecystitis	1 (2.2)	20 (29.0)	<0.001
Acute suppurative cholecystitis	25 (54.3)	48 (69.6)	
Acute gangrenous cholecystitis	16 (34.8)	1 (1.4)	
Perforation of gallbladder	4 (8.7)	0 (0.0)	

Data are indicated n (%) or $\bar{\chi }$ ± S.

**Figure 2:**
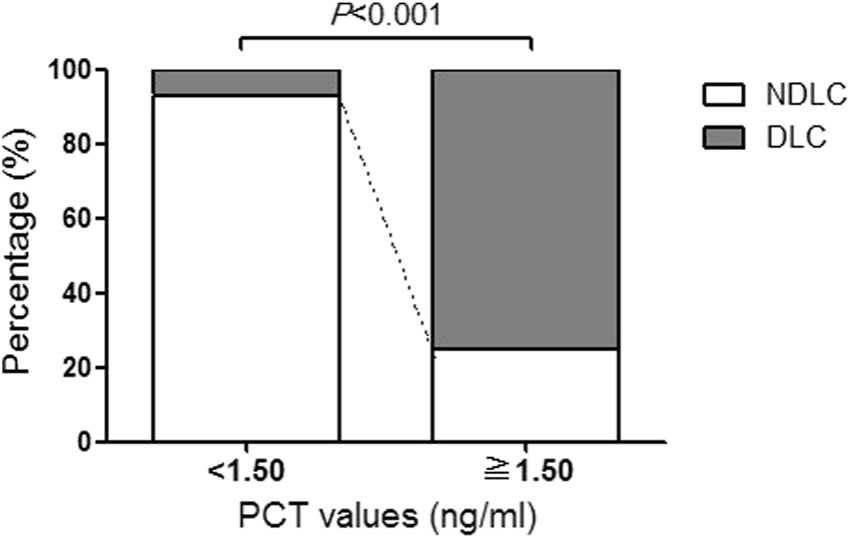
The bar charts showed the percentage of DLC and NDLC in subgroups, which were determined by PCT values. The PCT ≥ ≧ 1.5 ng/ml group had a significantly higher incidence of DLC (42/56, 75.0%) than the PCT < 1.5 ng/ml group (4/59, 6.8%) (*P* < 0.001).

To investigate the independent factor associated with DLC, the univariate and multivariate logistic regression models were performed. As indicated in Table 3, the significant variables in univariate results were patient’s BMI, CPR, PCT, peri gallbladder effusion, and cholecystolithiasis incarceration, these variables were further entered into a multivariate model. The only variable which was significant in both univariate and multivariate analyses was PCT (OR: 408.52, 95% CI: 41.17–4053.27, *P* < 0.001). The estimated odds ratio of PCT indicates a positive relation between PCT and the risk of DLC where higher the PCT may follow higher risk of DLC. To further investigate the cut-off, ROC analysis was used. The AUC of PCT was 0.927 (95% CI 0.882–0.973, *P* < 0.001). The cut-off suggested by comparatively maximum Youden’s index of PCT was 1.50 ng/ml with the sensitivity and specificity of 91.3% (95% CI 78.3–97.1) and 76.8% (95% CI 64.8–85.8), respectively. The patients with PCT ≥ ≧ 1.50 ng/ml (42/56, 75.0%) had significantly higher incidence of DLC than those with PCT < 1.50 ng/ml (4/59, 6.8%) (*P* < 0.001) (Fig. 2). The risk of DLC was significantly higher for patients with PCT ≥ ≧ 1.50 ng/ml (OR, 5.2 [95% CI, 3.7–7.5]; *P* = 0.004) than those with PCT < 1.50 ng/ml. Power analysis showed that the estimated power of the dichotomous PCT index was over 0.99, suggesting that the sample size was sufficient.

**Table 3:** Univariate and multivariate logistic regression models for DLC.

Parameters	Univariate		Multivariate	
	OR (95% CI)	P	OR (95% CI)	P
Age (year)	1.00 (0.97-1.02)	0.890		
Gender				
Male	ref.	—		
Female	1.39 (0.65-2.96)	0.395		
BMI (kg/m2)		0.024		0.186
<24	ref.	—	ref.	—
24-28	1.80 (0.75-4.28)	0.186	3.38 (0.84-13.66)	0.087
>28	5.45 (1.54-19.32)	0.009	3.57 (0.28-45.42)	0.327
WBC (10^9/L)	1.02 (0.87-1.20)	0.805		
CPR (mg/L)	1.02 (1.00-1.04)	0.025	1.01 (1.00-1.02)	0.151
PCT (ng/ml)	470.17 (59.65-3706.07)	<0.001	408.52 (41.17-4053.27)	<0.001
Peri gallbladder effusion				
No	ref.	—	ref.	—
Yes	6.12 (2.64-14.20)	<0.001	2.62 (0.62-11.06)	0.191
Cholecystolithiasis incarceration				
No	ref.	—	ref.	—
Yes	3.09 (1.35-7.06)	0.007	2.17 (0.51-9.19)	0.294
TG18 severity grading		0.201		
Grade I	ref.	—		
Grade II	2.47 (0.89-6.85)	0.083		
Grade III	1.50 (0.28-7.93)	0.633

**Figure 3:**
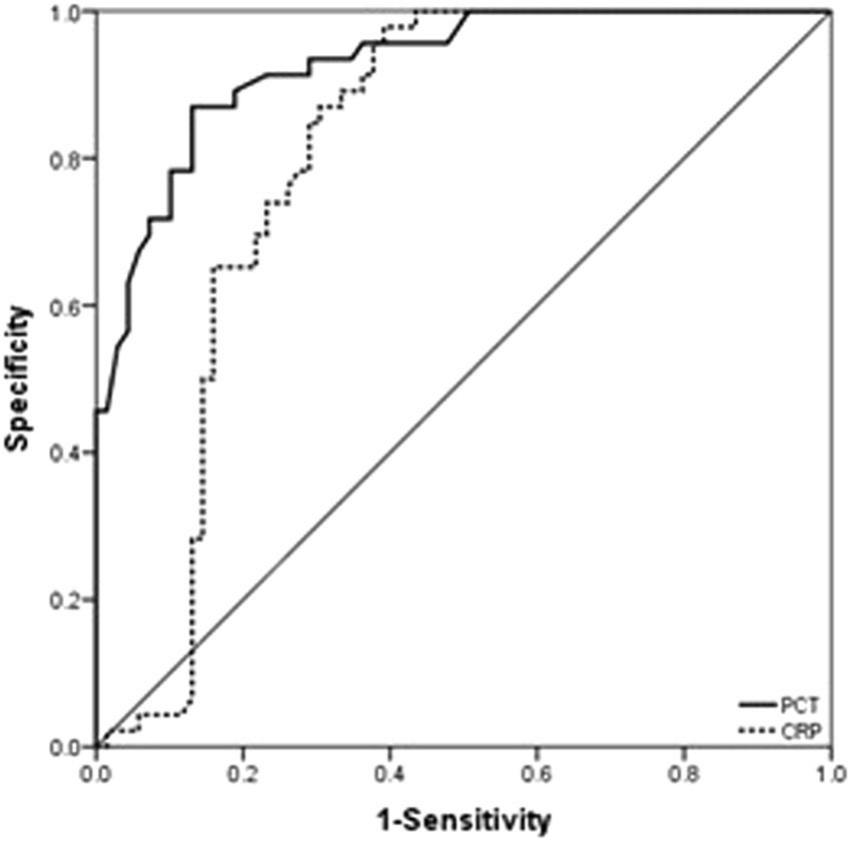
The ROC curve of PCT and CRP for analysis of difficult laparoscopic cholecystectomy for acute cholecystitis. Area under curve representing the diagnostic power, is 0.927 (95% CI 0.882–0.973, *P* < 0.001) for PCT and 0.806 (95% CI 0.723–0.888, *P* < 0.001) for CRP, respectively.

The ROC analysis was also conducted for the levels of CRP. The AUC of CRP was 0.806 (95% CI 0.723–0.888, *P* < 0.001). The cut-off suggested by comparatively maximum Youden’s index was 109.08 mg/L. The sensitivity and specificity were 80.4% (95% CI 65.6–90.1) and 71.0% (95% CI 58.6–81.0), respectively (Fig. 3). Power analysis showed that the estimated power of the dichotomous CRP index was over 0.99, suggesting that the sample size was sufficient.

## Discussion

According to TG18, LC is generally considered to be the first treatment option for mild to moderate AC, while cautiously recommended for the treatment of severe AC^1^. In severe AC, the risk of biliary tract injury and the conversion rate to open surgery will increase significantly due to severe edema and adhesion of the tissue^15^. Accordingly, it is often quite difficult to perform LC in patients with severe acute cholecystitis^3^. Predicting difficult cases of LC is important for the surgical treatment for AC. Ultrasonography is usually the first choice for the diagnosis of acute cholecystitis. It has been shown that the diagnostic sensitivity of ultrasonography for cholelithiasis is almost 98%, and the diagnostic value of ultrasonography for AC patients is about 54%-90%. However, the relationship between ultrasound findings and LC difficulty has not been investigated^16,17^. Similarly, computed tomography (CT) is of great diagnostic value for acute calculous cholecystitis, but of limited value in judging the severity of AC^4,7^. In terms of laboratory data, leukocyte counts in AC patients generally increase, but do not have a predictive function for the difficulty of surgery. Studies suggest that increased leukocyte count supports the diagnosis of AC, and increased leukocyte count is associated with increased postoperative complications^4,7^. In TG18, the increased leukocyte count is positively correlated with the severity classification of AC^8^. Similarly, CRP is a low-specific marker because it increases at 12 to 18 hours after tissue damage due to bacterial or viral infections. According to the Tokyo guidelines, CRP > 30 mg/L was one of the diagnostic criteria of AC. However, CRP levels were not used for determining the severity of AC^8^. On the contrary, some studies suggest that the serum level of CRP is positively related to the severity grading of cholecystitis. Beliaev *et al*.^18^ considered that CRP level has a better diagnostic ability in most AC patients with than leukocyte counts, and CRP is also a good diagnostic marker for the severity of AC. Díaz-Flores *et al*.^19^ even found that for the AC patient with CRP ≥ 110 g/L was positively correlated with the DLC. Our multivariate logistic regression analyses showed that CRP was not a predictor for DLC.

Surgeons often encounter a variety of unpredictable surgical difficulties in LC for AC patients, such as prolonged operation time, bile duct injuries, increased intraoperative bleeding or conversion to laparotomy^1^. Therefore, predicting the surgical difficulty is required for LC in AC patients. At present, there have been a number of studies on predicting the factor associated with the surgical difficulty of LC^2–5^. However, none of them reports a simple and feasible predictor for DLC. Identifying effective predictors of DLC can help to overcome the surgical difficulties encountered during LC. Consistent with the previous reports^9,10,19^, we observed an increase in WBC count, CRP and PCT levels as the disease progressed in AC, which may be attributed to AC-induced systemic inflammatory response. The PCT levels are directly proportional to the severity of the systemic inflammatory response. The persistent increase in PCT levels indicates that infection caused by AC was not effectively controlled or appropriately treated. Furthermore, TG18 considers that the difficulty of LC surgery depends largely on the severity of inflammatory reaction in the Calot triangle of AC patients^20^. We hypothesized that the difficulty in LC is associated with the degree of abdominal inflammatory response in AC patients. The increase of PCT level in AC patients is more likely to have DLC. In our patients, the AUC for the ROC curve of PCT values was 0.927, with a 95% CI 0.882–0.973 (*P* < 0.001). When the preoperative PCT value was equal to or greater than 1.50 ng/ml, DLC was more likely to occur. The sensitivity and specificity of the PCT for predicting DLC were 91.3% (95% CI 78.3–97.1) and 76.8% (95% CI 64.8–85.8), respectively.

In a prospective study, Patil and Inamdar assessed preoperative clinical data to identify predictor of the DLC^21^, and found that prior abdominal surgery, history of hospitalization, diabetes, obesity and gallbladder wall thickness was important predictors of DLC. Onoe *et al*.^22^ reported a preoperative scoring system for predicting the ability to achieve CVS of acute LC in AC patients. Multivariate logistic regression analysis showed that CRP > 55 g/L, cholecystolithiasis, duration of disease >72 h were the independent risk factors associated with the failure in achieving CVS. They have proposed a prediction scoring system for DLC including these three preoperative factors. However, they only assessed the ability to achieve CVS during LC. In our study, DLC was defined based on operative time, intraoperative complications and conversion rate of laparotomy. In addition, we found that different preditors for DLC, such as BMI, CRP, PCT and peri gallbladder effusion. Hayama *et al*.^3^ have reported that the gangrenous cholecystitis is considered as a risk factor for DLC in early surgical treatment for AC. For AC patients with delayed surgery, leukocyte count and age were positively correlated with the occurrence of the DLC and the conversion rate of open cholecystectomy. We did not analyze the histopathological findings, but compared the severity of AC based on TG18 grading. Most of the patients in DLC group had a higher TG18 grade *(P* = 0.002), including 36 (78.3%) patients with TG 18 Grade II and 4 (8.7%) patients with TG 18 grade III. The DLC group had a higher Clavien-Dindo grade in postoperative complications than the NDLC group (*P* = 0.024).

Some studies object to the inclusion of operative time in the definition of DLC, mainly because it relies on surgeon laparoscopic techniques^23^. To avoid these effects, all operations in our study were performed by the same surgeon with extensive experience in laparoscopic surgery. Most previous studies identified predictors of DLC based on the results of comparative analysis, which lacked authentic predictive models through ROC curve and AUC value analysis, along with sensitivity and specificity analysis of various factors. Bourgouin *et al*.^4^ reported a preoperative scoring system for planned and emergency operations, which included the following five preoperative variables: gender, history of cholecystitis, neutrophil count, fibrinogen and alkaline phosphatase to establish a risk assessment model with an area of 0.80 under the ROC curve. Our study found that in the PCT model analysis when considering PCT ≥ ≧ 1.50 ng/ml (*P* < 0.001) as predictors for DLC, the AUC value was 0.927 (*P* < 0.001). In addition, it is important to differentiate treatment according to the TG 18 grade since patients with different TG 18 grades may have varying pathophysiological processes and prognosis of AC. Nevertheless, our model still requires external validation. In addition, our study is limited by its retrospective nature and sample size. Also as a limitation, the power analyses in this study were post-hoc and should be calculated in advance for future works.

## Conclusions

Most DLC patients had higher preoperative TG18 grade and Clavien-Dindo grade of postoperative complications than the NDLC. This study shows that the PCT is superior to CRP in diagnosing DLC. The cut-off value for PCT of >1.50 ng/ml has a high predictive value for DLC in AC patients. Monitoring PCT trends may be used as a preoperative risk assessment in AC patients.
